# On proportional deformation paths in hypoplasticity

**DOI:** 10.1007/s00707-019-02597-3

**Published:** 2020-01-28

**Authors:** Erich Bauer, Victor A. Kovtunenko, Pavel Krejčí, Nepomuk Krenn, Lenka Siváková, Anna V. Zubkova

**Affiliations:** 1 Institute of Applied Mechanics, Graz University of Technology, Technikerstr. 4, 8010 Graz, Austria; 2 Institute for Mathematics and Scientific Computing, University of Graz, NAWI Graz, Heinrichstr. 36, 8010 Graz, Austria; 3 Lavrent’ev Institute of Hydrodynamics, Siberian Division of the Russian Academy of Sciences, Novosibirsk, Russia 630090; 4 Institute of Mathematics, Czech Academy of Sciences, Žitná 25, 115 67 Praha 1, Czech Republic; 5 Faculty of Civil Engineering, Czech Technical University in Prague, Thákurova 7, 166 29 Praha 6, Czech Republic; 6 Graz University of Technology, Rechbauerstr. 12, 8010 Graz, Austria

**Keywords:** 74C15, 74H40, 34D20

## Abstract

We investigate rate-independent stress paths under constant rate of strain within the hypoplasticity theory of Kolymbas type. For a particular simplified hypoplastic constitutive model, the exact solution of the corresponding system of nonlinear ordinary differential equations is obtained in analytical form. On its basis, the behaviour of stress paths is examined in dependence of the direction of the proportional strain paths and material parameters of the model.

## Introduction

The constitutive stress–strain relation for hypoplastic granular materials like cohesionless soil or broken rock is under our consideration. The respective constitutive law is of the rate type, incrementally nonlinear, and it is based on the hypoplastic concept proposed by Kolymbas [29]. Compared to incrementally linear constitutive equations, e.g. for hyperelastic and hypoelastic material laws, the hypoplastic constitutive equations are not differentiable at zero strain rate. This is due to different stiffnesses for loading and unloading typical for inelastic materials. In contrast to the classical elastoplastic concept, the strain in the theory of hypoplasticity is not decomposed into elastic and plastic parts. Detailed discussions about physical aspects of hypoplastic models can be found, for instance, in [20, 31, 36]; their response to cycling loading is studied in [9, 41], shear localization in [7, 10, 16, 23], critical states in [4, 5, 49], behaviour under undrained condition in [37], extension to a micropolar continuum in [23, 24], and thermodynamic aspects in [26, 42, 43].

Other typical representatives for incrementally nonlinear constitutive equations are, for instance, the Armstrong–Frederick model [2], the endochronic model by Valanis [45], the octolinear model by Darve [14, 15], the CLoE model by Chambon et al. [12], and the barodesy model by Kolymbas [30]. For variational approaches to modelling granular and multiphase media, we refer to [1, 27, 33].

Experiments with granular materials show a typical behaviour of the stress path obtained under a monotonic proportional deformation path which was formulated in two rules by Goldscheider [18]. The first rule says that a proportional deformation path starting from a nearly stress-free state results in a proportional stress path. The second rule says that a proportional deformation path starting from an arbitrary stress state asymptotically converges to the same proportional stress path as the one obtained for the initially stress-free state. These asymptotic properties of granular materials can be interpreted by fading memory of the material [21]. This feature is also confirmed by further experiments, e.g. [13, 44], and numerical simulations by the discrete element method, e.g. [25, 34].

As the asymptotic behaviour of the stress path for proportional deformation is an intrinsic property of granular materials, it has also great importance for constitutive models relevant to frictional granular materials. It can be noted that in many constitutive models the asymptotic behaviour is only fulfilled for particular deformation paths. The lack of this property can lead to large deviation of the prediction of stress paths not used for calibration.

In constitutive modelling, the asymptotic property is the starting point for barodesy modelling in [38], and it is also an intrinsic feature in hypoplasticity [20, 22, 28, 35, 36, 40]. With respect to a particular hypoplastic model by Kolymbas [28], stress paths were numerically investigated for initially axisymmetric stress states and axisymmetric proportional deformations. However, no general requirements for the asymptotic behaviour were formulated. A mathematical criterion providing a necessary condition for convergent asymptotic behaviour in incremental models was given first by Niemunis [40]. It requires that the normal distance of the generated stress path to the asymptote decreases with an increase in monotonic deformation, where the asymptote is obtained for the corresponding fixed strain rate starting from the initially stress-free state. The criterion by Niemunis is applicable to all rate-type models; however, it is restricted to proportional deformation paths with contractant volume strain behaviour.

In the present paper, a different strategy is proposed to examine the asymptotic behaviour using the analytical solution for the stress path, which depends on the direction of the proportional strain path, the initial stress state, and the material parameters involved in the constitutive equation. The main challenge in developing proper mathematical tools is the strongly nonlinear character of the underlying differential equations. Following the rate-independent technique developed in [11], we gave a rigorous mathematical proof of the existence of asymptotic states in [8, 32]. This idea is further developed in the present work.

To this end, a simplified version of the hypoplastic model by Gudehus [19] and Bauer [3] is considered. For the sake of simplicity, the pressure- and density-dependent properties of granular materials described in [3, 19] are omitted so that only two material parameters remain in the model. It can be noted that the same simplified version can also be obtained from the hypoplastic model by von Wolffersdorff [46] as shown in [6].

For the simplified hypoplastic model, we construct the solution of the corresponding nonlinear problem in a closed form. In this way, we make also a close link to barodesy models [30]. The exact solution allows us to describe analytically various scenarios of the behaviour of stress paths obtained from monotonic compression, extension, and isochoric deformations. The latter leads to stress limit states or the so-called critical stress states, which can be represented by a conical surface in the space of principal stresses. In particular, we identify the domain of the constitutive parameters which guarantee that for proportional deformation paths the corresponding stress paths starting from an arbitrary initial stress state are asymptotically stable.

## The model

We first fix some basic tensor notation. By ${\mathbb {R}}^{3\times 3}_\mathrm{sym}$, we denote the space of symmetric 3-by-3 tensors of second-order  and , which are endowed with the usual double dot product, the associated norm, and the trace, respectively:Here,  stands for the 3-by-3 identity matrix with the Kronecker symbol $\delta _{ij}=1$ for $i=j$, and zero otherwise. In physical interpretation,  corresponds to the Cauchy stress tensor, and  is the strain rate tensor. For time $t\ge 0$, we interpret  and  as time-dependent tensor-valued functions.

With respect to the normalized stress tensor , the general representation of the hypoplastic constitutive equation of the *Kolymbas type* can be written in the factorized form as the following tensor equation for the objective stress rate:1.1where  is a symmetric second-order tensor,  is a symmetric fourth-order tensor, and the double dot product is to be interpreted asThe dimensionless parameter $c<0$ scales the incremental stiffness and can be calibrated, for instance, based on an isotropic compression test. The right-hand side of (1.1) is a homogeneous function of degree one in . Note that dry granular materials are cohesionless, so that only negative principal stresses are relevant to the constitutive equation (1.1). Furthermore, we remark that particular representations of the tensor functions in (1.1) are based on terms from the general representation theorem of isotropic tensor-valued functions [47]. Various explicit versions are proposed in the literature (e.g. [3, 19, 29, 43, 48, 49]). In this paper, we consider a particular version of (1.1) proposed by Bauer in [5] in a simplified manner:1.2with the normalized stress deviator /3, where the symbol  stands for the fourth-order identity tensor, the symbol $\otimes$ denotes the dyadic product of tensors, and the term in (1.1) which is linear in  can also be represented as:1.3The constitutive constant $a>0$ is called limit stress state parameter and characterizes the shape of the conical limit stress surface or the so-called critical stress state surface in the principal stress space [5]. Critical states are defined for a vanishing stress rate under continuous isochoric deformation. For critical stress states, parameter *a* equals the norm of the normalized stress deviator, i.e. , and it can be related to the so-called critical friction angle [4]. While in the model by Gudehus [19] and Bauer [3] the value of *a* also depends on the orientation of the stress deviator, parameter *a* is assumed to be a constant in the present paper. For the granular friction angle $\phi \in (0,\pi / 2)$ such that $a =2\sqrt{2/3}\sin \phi / (3 -\sin \phi )$, we get the physical restriction $a <a_\mathrm{phys}=\sqrt{2/3}\approx 0.8165$ as $\sin \phi < 1$.

## Hypoplastic model under proportional deformation

Further we restrict ourselves to strain paths pointing in one fixed direction. Namely, we call a strain path *proportional* if there exists a time-independent symmetric second-order tensor  normalized by  and a scalar time-dependent function *s*(*t*) with $s(0) =0$ and $\dot{s}(t)>0$ such that it holds2.1In (2.1),  determines a prescribed direction in the space of symmetric second-order tensors, and *s* represents a monotonic increasing parameter. Moreover, we assume deformations for which the objective time derivative and the material time derivative coincide, i.e. , which, for example, holds for fixed directions of principal stresses. As the material behaviour described by Eq. (1) is isotropic, the strain rate tensor with fixed principal values, i.e. the strain increments are proportional and coaxial, corresponds to a stress rate tensor  with zero elements outside the diagonal. Thus, the relevant system of ordinary differential equations (ODE) reduces to three.

In this case, inserting the chain rule $\mathrm{d}/\mathrm{d}t = \dot{s} \mathrm{d}/\mathrm{d}s$ and  according to (2.1) and dividing with $\dot{s}\not =0$, we can rewrite the rate-independent relations (1) with respect to $s>0$ in the form:2.2or, equivalently, without factorization:2.3where the formula  was used, and2.4In the principal stress space, the representation of the normalized quantity  indicates the direction of the asymptotic stress state for $s \rightarrow \infty$. This observation will be specified below after the representation formula (5.6). Indeed, formula (5.6.1) shows that the evolution of  takes place in the 2D plane generated by constant tensors  and . We shall see that the scalar coefficient of  is dominant and determines the asymptotic convergence or divergence of the stress path as $s \rightarrow \infty$.

In system (2.3) of ordinary differential equations (ODE) for , the last term in the right-hand side is nonlinear in . For (2.3), we prescribe the initial condition at $s=0$:2.5where  is a given initial stress tensor.

In the following, we study the behaviour as $s\rightarrow \infty$ of solutions  to the nonlinear initial value problem (2) in dependence of the direction  of proportional strain paths, the material parameter *a*, and the initial stress .

### Motivating example: isotropic compression/extension

In this Section an assembly of grains with a grain skeleton is considered under compressive stresses. In mathematical terms, it means that all principal stress components are negative. Here, by compression or extension we mean the dynamics of the process defined by (2.1), where the strain rate can be, respectively, negative or positive.

*(i) Isotropic compression*: Under a monotonic displacement controlled isotropic compression, we understand the situation of Eq. (2.3) corresponding to the choice . Then  can easily be found as the solution of the linear scalar equation3.1that is,3.2Since the model is valid only for compressive stresses, we necessarily have . Furthermore, $c<0$ and $D^- <0$, so that the negative stress trace exponentially grows when the compression linearly increases along the direction of  for all $a>0$. Equation (2.3) turns into the form3.3In view of (3.2), the solution of (3.3) reads3.4If the deviator  of the initial stress is zero, that is, the initial stress is located on the isotropic axis generated by , then the whole stress path lies on the isotropic axis. Otherwise, if the initial stress deviator does not vanish, there are three possible scenarios for the evolution of  depending on whether $E^->0$, $E^- < 0$, or $E^- =0$.

*Scenario (a)* If $E^- > 0$, then we have $cE^- < 0$, and the stress path converges exponentially to the isotropic trajectory with increasing time, so that the model is exponentially stable with respect to small perturbations of the initial stress.

*Scenario (b)* The situation is totally different when $E^- < 0$. Then $cE^- > 0$, and small perturbations of the initial stress produce exponentially large deviation of the stress path from the isotropic trajectory.

*Scenario (c)* In the limit case $E^- = 0$, the distance of the solution trajectory from the isotropic axis remains constant.

Thus, scenarios (b) and (c) do not fulfil the second law by Goldscheider. According to the sign of $E^-$ in (3.3) we see that for the stability of the model with respect to variations of the initial stress (scenario (a)) it needs $a>a_{\min }$, and the critical parameter value is $a_{\min } = 1/(2\sqrt{3})\approx 0.2887$. For smaller values of *a* (scenario (b)), we are in the classical philosophical situation^1^ described by Frank Brentano.

**Fig. 1:**
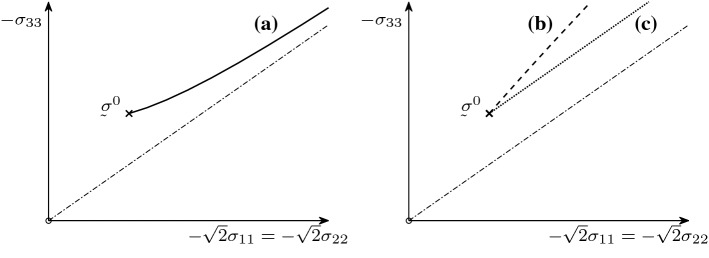
Stress paths obtained under monotonic isotropic compression and for different values of the parameter *a*

For illustration, in Fig. 1 we show the projection of the stress paths onto the so-called Rendulic plane for particular values $a=0.1$, $a=a_{\min }$, and $a=1$, and an initial stress . We observe that: for $a=1>a_{\min }$ (*solid curve*) according to scenario (a) the stress paths tend towards the isotropic axis (*dot dashed line*), which is, in this case, the asymptote; for $a=0.1 <a_{\min }$ (*dashed curve*), there is no asymptotic behaviour implying scenario (b); and for $a=a_{\min }$ (*dotted curve*), the stress path is parallel to the isotropic axis implying scenario (c).

*(ii) Isotropic extension*: For an initially prestressed state of monotonic isotropic extension, we have  resulting in the equations for :4.1and, respectively, for :4.2As given above, its solution can be written in the form4.3In this case, we have $D^+ >0$ and $E^+ > 0$, and the stress $\sigma (s)$ in (4.3) decays exponentially to zero as $s\rightarrow \infty$ independently of the choice of the initial condition.

We remark that according to the derived formulas (3) and (4) (as well as in the general case (2.3)) the parameter $c<0$ does affect neither the asymptotic states nor the shape of stress paths; rather, it influences how quick a proportional stress path is approached.

Motivated by this special example, it is our aim to investigate in the following the stress path under general proportional deformations.

### Analytical solution of the hypoplastic equation for general proportional deformations

The principal difficulty of solving the ODE (2.3) in the general form is its non-linearity in the stress. Here, we apply the following procedure consisting of three steps:

*Step 1*: We derive from (2.3) an ODE for the auxiliary scalar variable  and find its solution;
*Step 2*: Inserting the solution to Eq. (2.3) projected in the isotropic direction, we obtain a linear equation for  and solve it;
*Step 3*: Substituting this solution in the constitutive relation (2.3), we find an expression of  in the closed form.

A rigorous derivation of the analytical solution is given in the “Appendix.” Below we summarize the resulting formulas and discuss similarities between hypoplasticity and barodesy models.

Let  be as in (2.4) and assume . To simplify the formulas, we introduce the constants (see (17.1) and (17.6))5.1and the function *h*(*s*) with $h(0) =0$ and depending on the initial stress tensor , defined by the formula (see (16.4) and (17.3)):5.2In (17.1), we prove that the differential equation (2.3) can be transformed into the following equation:5.3It is worth noting that (5.3) has the structure (up to a factorization) of a constitutive relation adopted in *barodesy*; see, for example, [38]:5.4where  is a given direction of the proportional stress path and ${\mathfrak {f}}$ and ${\mathfrak {g}}$ are model parameters. The exponential expression of  is formal and admits the expansion in $-\alpha$:5.5The first two linear asymptotic terms in (5.5) with $\alpha =-3a$ yield exactly  in (2.4), and the nonlinear behaviour of  with respect to  is substituted in the Kolymbas-type hypoplastic model by the norm  (which is normalized to one here). In this context, it can be mentioned that with constant *a* Eq. (1) models the stress limit condition by Drucker–Prager. For a refined modelling of the stress limit condition for granular materials, factor *a* should depend on the orientation of the stress deviator, i.e. it should be a function of the so-called Lode angle, which allows the adaptation of arbitrary conically shaped stress limit conditions in the principal stress space as outlined in [3, 4]. Taking into account the contribution  in Eq. (5.5), the barodesy model describes a stress limit condition close to the one by Matsuoka–Nakai [39] as shown in [17, 38]. In hypoplasticity, the stress limit condition by Matsuoka–Nakai can be predefined as shown, for instance, in [4, 46]. In both models (barodesy and hypoplasticity), parameters $\alpha$ and *a* are related to the granular friction angle.

Based on the representation (5.3) which is linear in , the solution of the initial value problem (2) can be found in the closed form (see (18.3)):5.6with *E* and *D* defined as in (5.1), *h*(*s*) as in (5.2), and  as in (2.4), whereas . For the special case  , that is,  for $a\not =0$, see formulas (19). Due to , from (5.6) we compute  and the normalized stress tensor5.7Therefore, $D-E<0$ in (5.7) proves the asymptotic convergence5.8exponentially as $s \rightarrow \infty$.

Since only negative principal stresses are relevant for the underlying model, it is important to discuss their feasibility. For this reason, we define the *feasible cone*:6where $\sigma _1$, $\sigma _2$, and $\sigma _3$ are eigenvalues of . Note that . Therefore, if $E\ge D$, then $\exp (c E s) \le \exp (c D s)$ (noting that $c<0$), so that rearranging the terms in Eq. (5.6) equivalently as5.6.1the factors in front of  and  are positive. Assuming that  and choosing the initial stress state  thus guarantee that the whole stress path  is contained in ${\mathcal {K}}_\mathrm{f}$ for all $s>0$. The detailed investigation of necessary and sufficient conditions on *a* and  to ensure that  reached by (2.4) lies in ${\mathcal {K}}_\mathrm{f}$ is the subject of the forthcoming research.

In the next Sections, we investigate the asymptotic behaviour as $s\rightarrow \infty$ of stress paths (5.6) in dependence of specific deformations .

## Asymptotic behaviour of stress paths under various proportional deformations

Similarly to the motivation example, we should distinguish contractant (volume-decreasing) from dilatant (volume-increasing) states. For this task, we call by *contractant* the compression corresponding to , and by *dilatant* the extension when , while  corresponds to the volume-preserving deformation.

To this end, we note that paths with  will eventually approach the origin as they are proportional volume-increasing deformation paths. However, there are examples of axial loading paths, e.g. , with . Mašín [34] also observed with discrete element method simulations the so-called asymptotic extension states, that are obtained by  with axial loading.

**Fig. 2:**
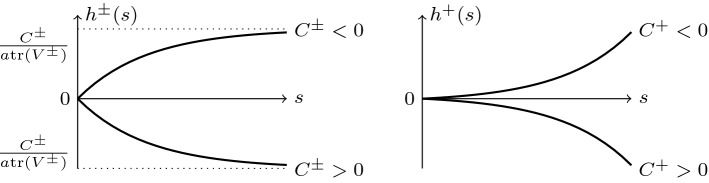
Sketch of the asymptotic behaviour *h*(*s*) for $\mathrm{tr}(V^\pm )<0$ (left) and $\mathrm{tr}(V^+)>0$ (right)

*(i) Contractant straining*: Let a tensor  be prescribed such that . Recalling , we have  for all $a>0$. According to (5.2), it follows  and the finite limit (see Fig. 2 the left plot):7.1

From (5.1), we infer $D^- <0$; hence, the second term in the right-hand side of (5.6) grows exponentially along . The asymptotic behaviour of the first term depends on the sign of $E^-$: it grows to plus or minus infinity if $E^-<0$ and decays to a finite number if $E^- =0$ and to zero if $E^- >0$. The latter case corresponds to the proportional contractant deformation such that:7.2This condition is equal toand describes the *asymptotically stable* stress path  in (5.6) attracting the direction of  as $s\rightarrow \infty$ by the mean of descent distance:7.3We remark that the decay of the distance in (7.3) holds only for sufficiently large values of *s*. For instance, the distance may increase in some interval $s\in (0,s_0)$ with $s_0>0$ satisfying $c E^- s_0 +h^-(s_0) =0$, when $c E^- <0$ and $h^-(s_0) >0$ for $C^-<0$ in (7.1).

*(ii) Dilatant straining*: Let  obey . We consider first the case of  and similarly to (7.1) (see Fig. 2 the left plot) obtain8.1Therefore, the stable asymptotic behaviour under the proportional dilatant deformation is guaranteed by $D^+ >0$ and $E^+ >0$, that is:8.2or, equivalently,then the stress path  in (5.6) *decays asymptotically* to zero:8.3By this, if $E^+ >D^+$, then the stress path is closer to ; otherwise, it is closer to  when the opposite inequality $E^+ <D^+$ holds.

In the *special case* of , that is, , from (19) we have9The asymptotic stability should be considered separately.

If , then  and follows the limit in (5.2) (see Fig. 2 the right plot):10.1In this case, $\exp (c E^+ s +h^+(s))$ and $\exp (c D^+ s +h^+(s))$ entering the stress path  in (5.6) may obey various asymptotic behaviour in dependence of the sign of parameter $C^+$ in (10.1) and the values of $E^+$, $D^+$ in (5.1). The *asymptotically stable* decay to zero of stress paths is described by the following two cases: either $C^+ =0$ (hence, $h^+(s)\equiv 0$) and $E^+ >0$, $D^+ >0$, that is:10.2or $h^+(s)\rightarrow -\infty$ provided by $C^+ >0$:10.3In the both cases (10.2) and (10.3), the exponential convergence holds:10.4The non-monotone behaviour of  is admissible for dilatant, too.

*(iii) Volume-preserving deformation* Consider a proportional deformation  with ; then, , $E=a$, and $D =0$ in (5.1); hence,with , and the limit:11.1We note that the limit states  in (11.1) for all admissible directions  with  and initial stresses  form the cone of the limit stress states with the apex in the origin of the space of principal stress axes. From (11.1), we find that ; thus, the interaction of this cone with the deviator plane, i.e. the plane with  (see [4]), is represented by the circle of radius *a*:11.2The analysis of asymptotic behaviour of stress paths under various proportional deformations from Sect. 4 in dependence of the parameters is summarized for convenience in Table 1.

**Table 1:** Domain of parameters for asymptotically stable stress paths

	Asymptotically stable growth/decay			Attracting
Contractant	$D^- <0$, $E^- >0$			
Dilatant		$D^+ >0$, $E^+ >0$		$E^+ >D^+$
		$D^+ >0$, $E^+ >0$	$C^+=0$	
		$$C^+>0$$		

## Discussion of the asymptotic results

In the following, we omit the superscripts $-, +$ and study how the parameters *D* and *E* defined in (5.1) are connected with the choice of *a* and . Indeed, from (2.4) we have ; hence, , and we get that *D* and *E* are only dependent on *a* and . We note that the range of  is limited by . To prove this, we considered ; therefore,  becomes extreme if $U_{ij}=0, i\ne j$, so only the entries on the diagonal of  are distinct from zero. With the arithmetic quadratic mean inequality, we get:and equality holds for $U_{11}=U_{22}=U_{33}=1/\sqrt{3}$.

**Fig. 3:**
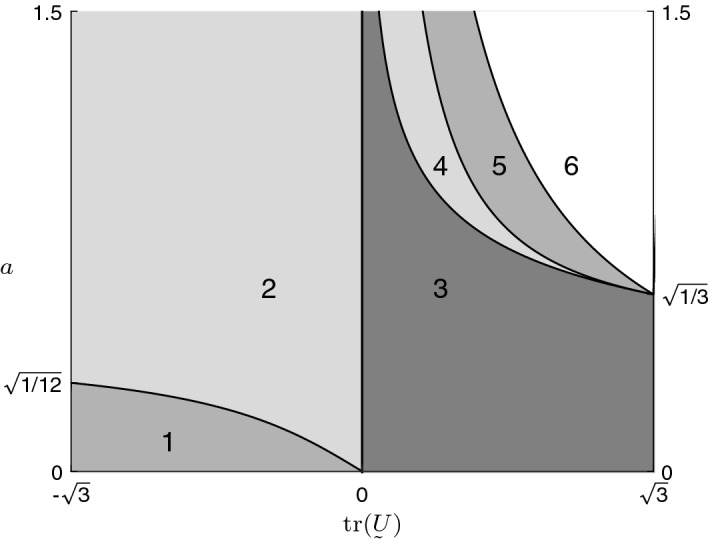
Visualization of the domain of parameters

As we see in Table 1, the relations of *D* and *E*, one to each other and to zero, are crucial for the asymptotic behaviour of  as $s\rightarrow \infty$. Depending on  (x-axis) and *a* (y-axis), these relations are plotted in Fig. 3.

Here, we consider the formal description of the plot, which means the equations of the curves separating the different greyscale areas, starting in the down left corner:

**–**: between area 1 and area 2:
**–**: between area 2 and area 3:
**–**: between area 3 and area 4:
**–**: between area 4 and area 5:
**–**: between area 5 and area 6:

The meaning of the corresponding areas is the following:

$$\square $$
in the light grey areas 2 and 4 (where parameters $D<0<E$), stress paths are going to infinity attracting ;
$$\square $$
in the medium grey areas 1 and 5 (where $D<E<0$), stress paths do not have any asymptote and diverge;
$$\square $$
in the dark grey area 3 (where $0<D<E$) stress paths go to zero attracting ;
$$\square $$
in the white area 6 (where $0<E<D$), the behaviour of the stress paths depends also on the initial stress state , while in the other areas the asymptotic behaviour is independent of the initial stress state.

Further we illustrate the usage of Table 1 for the cases of contractant, dilatant, and volume-preserving deformations.

*(i) Contractant straining*

If , the stress path is asymptotically stable in area 2. We get the lower bound for , which means isotropic compression, by

$$\begin{aligned} a>a_{\min } =\frac{1}{2\sqrt{3}}\approx 0,2887. \end{aligned}$$

For $a>a_{\min }$, we can be sure to have asymptotically stable behaviour for arbitrary proportional deformation (see Fig. 1 from Sect. 3.1).

*(ii) Dilatant straining*

If , the stress path is asymptotically stable in areas 3 and 4: in area 3, the path tends to zero, while in area 4, it tends to infinity. We get the upper bound for , which means isotropic extension, by

$$\begin{aligned} a<a_{\max } =\frac{1}{\sqrt{3}} \approx 0,5774. \end{aligned}$$

For $a<a_{\max }$, we have asymptotically stable behaviour for arbitrary proportional extension; in this case, the stress path will also tend to zero.

*(iii) Volume-preserving deformation*

For the limiting case of , the stress path will tend to a certain stress state at the critical cone. To illustrate the matter, we plot few results of numerical simulation.

**Fig. 4:**
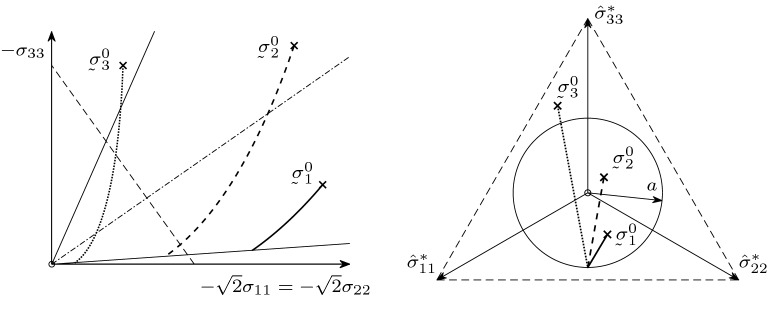
Stress paths for different  under volume-preserving deformation

In the left plot of Fig. 4, the stress paths are projected in the plane spanned by $-\sigma _{33}$ and the first median of the $-\sqrt{2}\sigma _{11}=-\sqrt{2}\sigma _{22}$-plane. The dot dashed line is the isotropic axis; the outer solid lines are the intersection with the critical cone. In the right plot, the paths are projected onto the deviator stress plane . In the deviator plane, the cutting line with the critical cone forms a circle of radius *a* according to (11.2). In Fig. 4, we see three example stress paths under volume-preserving deformation  with the parameter $a=0.35$ and three initial stress statesWe can observe that for volume-preserving deformation all stress paths tend to the critical cone independent of the choice of .

**Fig. 5:**
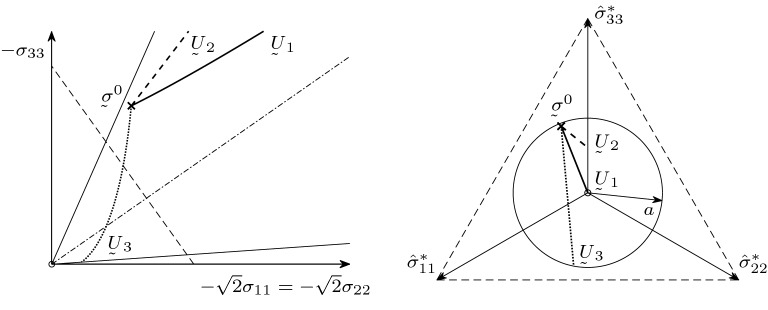
Stress paths for different

For comparison, in the similar coordinate axes as in Fig. 4, in Fig. 5 we see three stress paths starting at the initial stress state  with the parameter $a=0.35$ under three different deformationsWe see that for different deformations  we get different behaviour of the stress paths. For  (solid curve), we have isotropic compression, and the corresponding stress path tends to the isotropic axis. For  (dashed curve), we have uniaxial compression, and we can also observe an asymptotic behaviour. For  (dotted curve), we have volume-preserving deformation and the stress path goes to the critical cone.

## Case studies

In this Section, we will discuss the case of isotropic deformation and uniaxial deformation in more detail.

### Isotropic deformation

*(i) Compression*: We can now give a more detailed interpretation of the results of Sect. 3.1. The contractant stress path (3.4) with $D^- =-(3 a^3 +1/ \sqrt{3})/ (\sqrt{3}a +1)$, $E^- = 2a - 1/ \sqrt{3}$ is exponentially stable along  for $a >1/ (2\sqrt{3})\approx 0.2887$ and $a<a_\mathrm{phys}$, and its distance from the -axis grows to infinity if $a < 1/ (2\sqrt{3})$ and remains constant for $a = 1/ (2\sqrt{3})$.

*(ii) Extension*: In the dilatant case (4.3) with $D^+ =\sqrt{3} a^2 + a + 1/ \sqrt{3}$, $E^+ = 2a + 1/ \sqrt{3}$, , the critical value is $a_{\max } =1/ \sqrt{3} \approx 0.5774$. Then  and similarly as in formulas (19.2) and (19.3) we obtain the solution , which agrees with (4.3) for $D^+ = E^+ =\sqrt{3}$. This corresponds to cases (8.2), (9), and (10.2), and from (4.3), we conclude unconditional asymptotic decay to zero:12By this, if $E^+ >D^+$, which holds for $a<1/ \sqrt{3}$, then  attracts closer the direction of . Otherwise,  is more attractive. The result in dependence on the parameter *a* is gathered for convenience in Table 2.

**Table 2:** Stress path under coaxial deformation

*a*	$\bigl (0,\frac{1}{2\sqrt{3}}\bigr ]$	$\bigl (\frac{1}{2\sqrt{3}},\frac{1}{\sqrt{3}}\bigr )$	$\frac{1}{\sqrt{3}}$	$\bigl (\frac{1}{\sqrt{3}},a_\mathrm{phys}\bigr )$
Compression	Growth:			
	Unstable	Monotone asymptotically stable attracting		
Extension	Monotone asymptotically stable decay:			
	Attracting			Attracting

We remark that this particular case of isotropic compression/extension implies $\mu =D^\mp$ being the single eigenvalue and $\mu =E^\mp$ the double eigenvalue for the following eigenvalue problem written with respect to $\mu$, respectively:13where  stands for the 3-by-3 matrix of ones and  is the eigenvector corresponding to $D^\mp$; see [32] for details.

### Uniaxial deformation

*(i) Asymptotic compression*: We consider a so-called oedometric test, which is a uniaxial deformation under lateral zero strain, i.e. , then , , and . Substituting it in (5.1) gives $E^- =(2a^2 +a -1/3)/ (a+1)$, $D^- =-(a^3 +1/3)/ (a+1) <0$, and in (5.2) provides:14The condition (7.2) for $E^- >0$ implying $2a^2 +a -1/3 >0$ holds true if $a >a_0 :=(\sqrt{11/3} -1)/ 4\approx 0.2287$; then, it provides the asymptotically stable stress path along the direction of  as described in (7.3), which is monotone if $C^-\ge 0$. Otherwise, if $0 <a \le a_0$, then the stress path grows to infinity.

*(ii) Asymptotic extension*: For  and  such that , and , we calculate $E^+ =(-2a^2 +a +1/3)/ (1 -a)$, $D^+ =(a^3 -1/3)/ (1 -a)$, and15Both conditions (8.2) are valid for $a <a_1 :=1/ \root 3 \of {3} \approx 0.6934$ providing $a^3 <1/3$, since $a_1 <a_3 :=(\sqrt{11/3} +1)/ 4\approx 0.7287$ for $2a^2 -a -1/3 <0$. This guarantees the asymptotic decay of the stress path to zero as described in (8.3). By this, $E^+ >D^+$ implying $a^3 +2a^2 -a -2/3 <0$ holds for $a<a_2\approx 0.7131$; then,  attracts closer the direction of ; otherwise,  is more attractive when $E^+ <D^+$. For $a =1$, the condition (9) is satisfied since $a >1/ \sqrt{3}$. For $a>1$, the conditions (10.2) and (10.3) for stable decay can be realized only for special initial states . In all other cases, the stress path grows to infinity. Since $a_\mathrm{phys} <1$, we summarize the result in Table 3.

**Table 3:** Stress path under uniaxial deformation

*a*	$(0,a_0]$	$(a_0,a_1)$	$[a_1,a_\mathrm{phys})$
Compression	Unstable growth	Asymptotically stable growth attracting	
Extension	Asymptotically stable decay attracting		Unstable growth

## Conclusions

In the paper, for a constitutive model based on the concept of hypoplasticity of the Kolymbas type the stress paths obtained under proportional deformations are investigated. In particular, a simplified hypoplastic constitutive equation is considered where the objective stress rate is a function of the current stress and strain rate. The model is obtained by omitting the influence of the change in the pressure-dependent relative density in the hypoplastic model originally proposed by Bauer and Gudehus. For arbitrary proportional deformations starting from the stress-free state, the corresponding stress paths are linear which is in accordance with the first law by Goldscheider and also an important property for constitutive models relevant to frictional granular materials. From experiments, it is known that significant changes in the density of the granular material can lead to a slightly curved stress path which can also be influenced by grain crushing under higher stresses. Such properties can be taken into account using more enhanced hypoplastic models, which are not considered in the present paper.

The hypoplastic equation considered only includes two constitutive constants: a stiffness parameter and a limit stress state parameter. The former can be related to an isotropic compression test and the latter to the so-called critical friction angle defined in the steady state of a cohesionless granular material under triaxial compression. Although the influence of the direction of the stress deviator on the limit stress state parameter is neglected, it does not mean a restriction of the general results drawn. A relevant relation between the values for the direction of the stress deviator of triaxial compression and the one for another direction can, for instance, be obtained by interpolation.

For the hypoplastic constitutive equation considered, the analytical solution of the stress paths depending on monotonous linear deformation paths is obtained in a closed form. On its basis, the course of the stress paths is examined in dependence of the material parameters and prescribed proportional contractant, dilatant, and volume-preserving deformations. It is shown that stress paths starting from arbitrary initial stress states are usually nonlinear, and their convergence to a proportional stress path (second law by Goldscheider) is asymptotically stable only for a certain domain of the limit stress state parameter, which is related to the critical friction angle.

## Appendix

We start with the following consequences of formula (2.3).

*Step 1*: The double dot product of (2.3) with  and recalling  leads to:

16.1

The double dot product of (2.3) with  and using  leads to:

16.2

For  and , subsequently dividing (16.1) with , multiplying (16.2) with , subtracting them and using , this results in the linear differential equation:

16.3

which admits the exact solution expressed in the following analytical form:

16.4

*Step 2*: We insert (16.4) in (2.3) such that it becomes linear in :

17.1

In the sequel, it will be useful to introduce an auxiliary function such that

17.2

that is:

17.3

Then (17.1) can be rewritten equivalently as

17.4

The double dot product of (17.4) with  leads to a linear differential equation for :

17.5

which admits the exact integral depending on parameter *s*:

17.6

*Step 3*: We plug (17.2), (17.4), and (17.6) in the following differential quotient:

18.1

and after its integration, we derive the solution in the closed form:

18.2

or, equivalently, as the sum of two exponential functions:

18.3

The above consideration holds true for . Otherwise, if  implying  for $a\not =0$, then (16.3) follows the solution:

19.1

Consequently, with the function

19.2

from (16.2) we find , and similarly to (18.1), this follows the linear equation  and its solution:

19.3

## Footnotes

1 What is at first small is often extremely large in the end. And so it happens that whoever deviates only a little from truth in the beginning is led farther and farther afield in the sequel, and to errors which are a thousand times as large (F. Brentano, On the Several Senses of Being in Aristotle. Herder-Verlag, Freiburg, 1862).
